# Supplementary material for: Genetic Diversity and Relationships of Listeria monocytogenes Serogroup IIa Isolated in Poland
Source: Microorganisms. 2022 Feb 28;10(3):532. doi: 10.3390/microorganisms10030532 (PMC8951407; doi:10.3390/microorganisms10030532)
Supplement: Supplementary file 1 [file microorganisms-10-00532-s001.zip › Supplementary files/Figure S1.pdf]

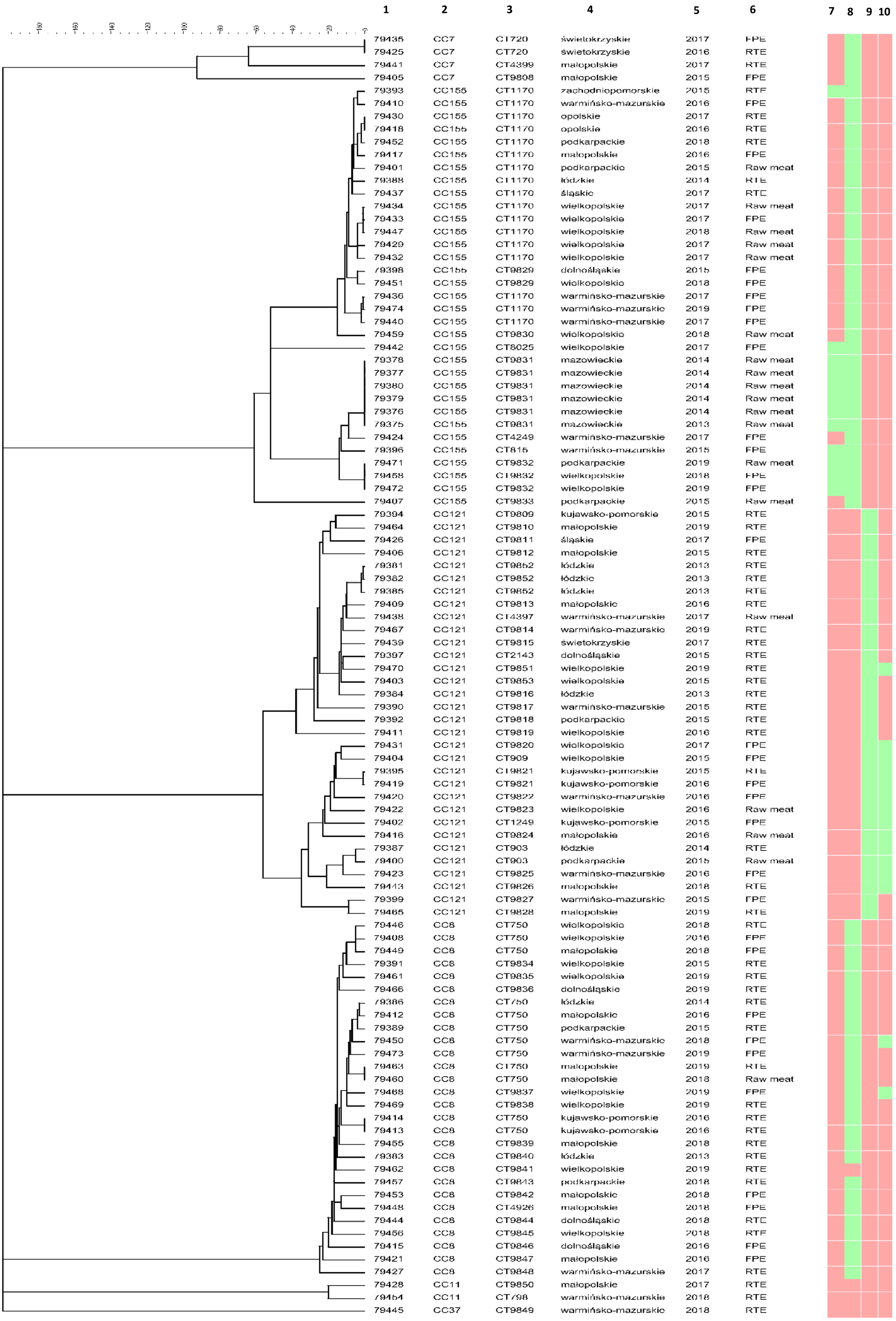

Figure S1. Phylogenetic tree of all 100 *L. monocytogenes* isolated tested based on the cgMLST analyses and prevalence of the selected genes (green – gene presence, red – gene absence). Column designations: 1. Isolate ID number, 2. Isolate CCs, 3. Isolate CTs, 4. Isolate source (voivodeship), 5. Year of isolation, 6. Isolate origin, 7. *bcr*ABC genes, 8. SSI-1 genes, 9. SSI-2 genes, 10. *ermC* (*Tn*6118_qac) gene.
