# Supplementary material for: Genetic Diversity and Relationships of Listeria monocytogenes Serogroup IIa Isolated in Poland
Source: Microorganisms. 2022 Feb 28;10(3):532. doi: 10.3390/microorganisms10030532 (PMC8951407; doi:10.3390/microorganisms10030532)
Supplement: Supplementary file 1 [file microorganisms-10-00532-s001.zip › Supplementary files/Figure S2.pdf]

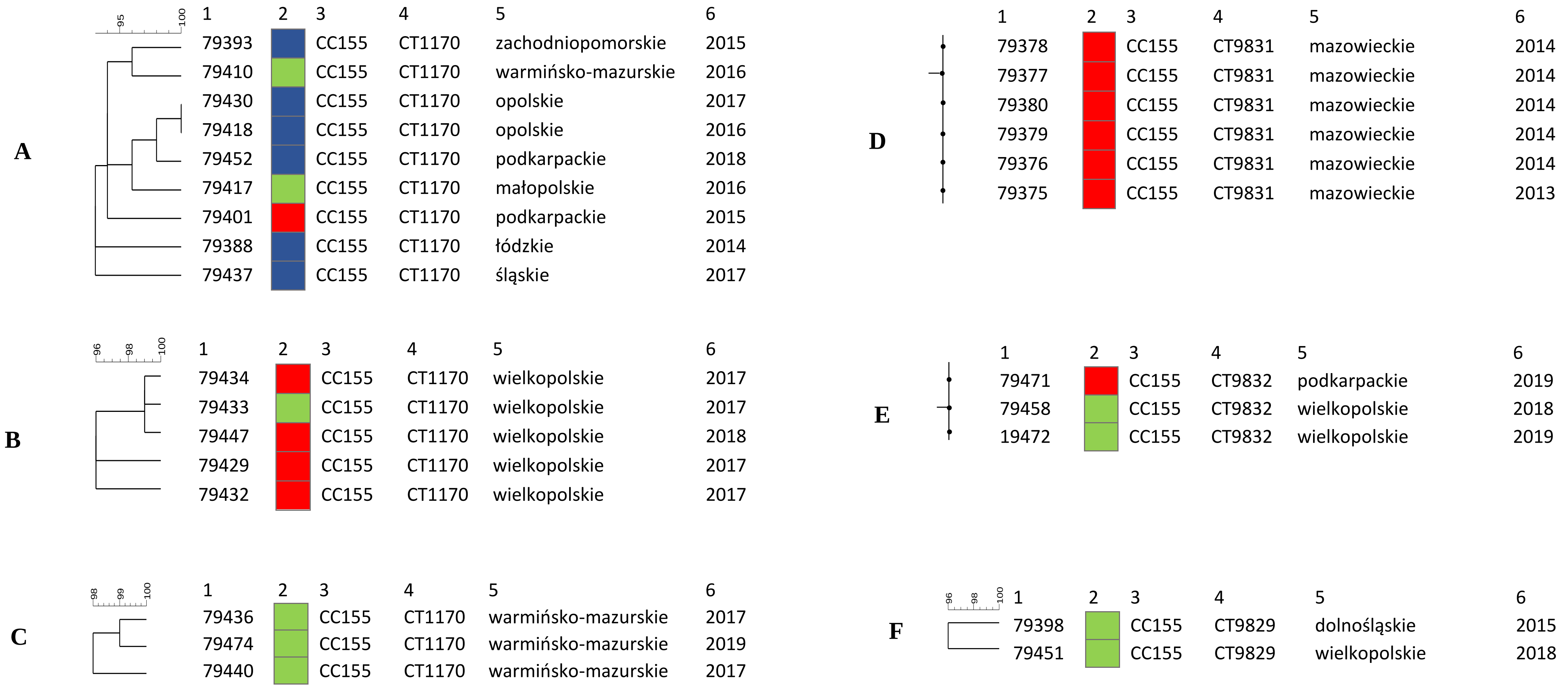

Figure S2. Comparison of *L. monocytogenes* CC155 isolates showing over 93% similarity of macrorestriction profiles obtained with the cgMLST method. Column designations: 1. Isolate number, 2. Isolate origin (blue – ready-to-eat food, green - food production environments, red – raw meat), 3. CC number, 4. CT number, 5. Isolate source (voivodeship), 6. Year of isolation.
